# Editorial: Environmental factors in autoimmunity

**DOI:** 10.3389/fimmu.2023.1361884

**Published:** 2024-01-16

**Authors:** Shannon E. Dunn, Jorge Correale, Jennifer L. Gommerman, Marc S. Horwitz

**Affiliations:** ^1^ Department of Immunology, University of Toronto, Toronto, ON, Canada; ^2^ Institute of Biological Chemistry and Physiocochemistry, School of Pharmacy and Biochemistry, University of Buenos Aires, Buenos Aires, Argentina; ^3^ Department of Microbiology and Immunology, University of British Columbia, Vancouver, BC, Canada

**Keywords:** environmental factors, autoimmunity, microbiome, diet, viruses

While the T and B lymphocyte repertoires are designed to be able to recognize foreign antigen (pathogens) to protect the host, significant portions of these repertoires can recognize self-antigens. Normally this is not a problem for the host because such autoreactive lymphocytes are removed (clonal deletion), redeemed by B cell receptor (BCR) editing, anergized, or actively suppressed in the periphery by regulatory T and B cells (1, 2). Despite these mechanisms, an estimated 7-9% of humans are diagnosed with an autoimmune disease (3). Puzzlingly, an equal percentage of healthy people have autoreactive anti-nuclear antibodies in their blood despite having no clinical evidence of autoimmunity (4). Classic studies that reported on the concordance of autoimmunity in monozygotic and dizygotic twins indicated that a large fraction of autoimmune risk (>50%) is conferred by environmental factors that interact with genetic factors to initiate disease (5). Thus, autoimmunity may be largely preventable if the specific causal environmental factors are identified, as seminal studies have shown that initial autoimmune pathogenic events take place prior to clinical manifestations.

This Research Topic aimed to provide new insights into environmental risk factors in autoimmunity and includes reviews and articles that describe how dietary factors and obesity, maternal and early life factors, viruses, the microbiome, and inhaled environmental toxins influence the development of autoimmunity.

## Dietary factors and obesity in autoimmunity

Past studies have identified obesity as a common risk factor for many autoimmune diseases including multiple sclerosis (MS), systemic autoimmune erythematosus (SLE), and Alopecia Areata (AA) (Touil et al.; Correale and Marrodan). Correale and Marrodan reviewed how the adipose tissue becomes inflamed with obesity and how this leads to the development of pro-inflammatory adipokine production that can enhance neuroimmune mechanisms in MS and in animal models of MS. Touil et al. added to this theme by reviewing how other lifestyle factors including diet quality and dietary deficiencies potentially contribute to autoimmunity development. The general conclusion from this review was that consumption of a diet rich in vegetables, dietary fibers, and polyunsaturated fatty acids (including fish oils) and low in saturated fat is protective against autoimmunity. This review also cited that deficiencies in vitamin D and folate are common in autoimmune disease.

A difficulty with interpreting association studies is the possibility of reverse causality–that the disease modulates exposure to the environmental factor. Mendelian randomization (MR) is a genetic approach whereby the presence of small nucleotide polymorphisms (SNPs) that associate with environmental exposure are compared between disease cases and controls. Since inheritance of the SNP influences biology from conception, causality can be inferred in this analysis. Using MR, Yang et al. found that genetically-predicted folate levels inversely associated with development of the autoimmune skin disease Vitiligo (odds ratio=0.47, P=1.3 x 10^-4^), but not with development of inflammatory bowel disease (IBD), rheumatoid arthritis, or SLE. This finding will direct future efforts to unravel the biological mechanisms of how folate is protective against autoimmunity in Vitiligo.

As overviewed by Bugbee et al., dietary factors can also modulate the microbiome to shift the immune system towards a state of tolerance through activation of the IL-10 receptor, a key cytokine signaling pathway that for the most part down-regulates inflammation, but with some important (and puzzling) exceptions. Alternatively, the diet can provide a source of epitopes that can sustain cross-reactive autoreactive T and B cell responses. Specifically, Chunder et al. discussed how MS patients have greater IgG reactivities to bovine and goat milk compared to healthy controls: greater IgG reactivities were not present against sheep or plant-based milk in MS patients. IgG antibodies enriched in MS were reactive against the milk proteins casein and β-lactoglobulin; those patients having the highest reactivities to β-casein exhibited the highest disability scores. Past work by this group demonstrated molecular mimicry between casein and the myelin antigen protein, myelin-associated glycoprotein (6). Together these findings raise the prospect that anti-milk IgGs may be pathogenic in MS and that plant-based milk products could be novel dietary interventions for this disease.

## Exposure to mercury and other inhaled pollutants and autoimmunity

Kawasaki disease (KD) is an autoimmune/autoinflammatory disease that involves activation of the inflammasome and targets the coronary arteries in children. Past studies have described “outbreaks” of KD in Japan in years when wind-patterns shifted, bringing more pollutants from China (7). Intrigued by this and case reports of KD developing in response to exposure to mercury vapors (8), Alphonse et al. examined the effect of mercury on inflammasome activation in the presence of *Lactobacillus* cell wall extract (LCWE), which is a factor that can induce KD-like disease in mice. They found that mercuric chloride raised calcium levels in bone-marrow derived DCs, providing signal 2 for NLRP3 inflammasome activation in these cells. Furthermore co-administration of mercuric chloride with LCWE markedly increased serum levels of pro-inflammatory cytokines IL-1 and IL-18 and enhanced the incidence and severity of coronary arteritis in mice. These findings provide proof of concept that mercury may be an environmental contributor to KD.

Inhalation of silicon dioxide (cSiO_2_) and cigarette smoke has been linked to the development of SLE and MS (Touil et al.; 9). In this Research Topic, Heine et al. demonstrated that intranasal instillation of cSiO_2_ in lupus prone, New Zealand Black/White F1 (NZB/W F1) mice induced the development of ectopic lymphoid structures (ELS) in the lung and accelerated the development of lupus nephritis. Provision of clinically-relevant doses of prednisone in the diet reduced cSiO_2_-induced pulmonary ELS formation, nuclear-specific autoantibody production, and glomerulonephritis, but did not increase animal survival due to treatment-associated toxicity. These experiments highlight a role of lung inflammation in SLE and cSiO_2_-accelerated SLE as a model for testing existing and novel therapies for SLE.

## Microbiome and viruses in autoimmunity

Articles in this Research Topic also reported on the role of viruses and the microbiome in the development of autoimmunity. A review by Strauchan et al. investigated how maternal factors such as the maternal microbiome, breast-milk-derived autoantibodies may protect against the development of type I diabetes (TID), whereas, an article by Yue et al. described how the host genotype, specifically a high-risk major histocompatibility haplotype can shape the microbiome and serum metabolites in TID patients. Another MR study addressed the bi-directional relationship between genetic SNPs predictive of IBD and the development of herpesvirus-associated diseases, chicken pox, herpes zoster and mononucleosis. Despite prior case control studies that reported associations between these infectious diseases caused by varicella-zoster virus and Epstein Barr virus (EBV) and IBD, this MR study did not find a causal association. The authors did find that IBD-associated SNPs predicted mononucleosis, thereby suggesting that genes that are associated with autoimmunity may predispose to a more pro-inflammatory manifestation of EBV infection.

Enteroviruses, including coxsackieviruses are over-represented in children that progress to develop TID and coxsackievirus infection has been shown to accelerate the development of TID in both female and male non-obese diabetic (NOD) mice (10, 11). Here, an article by Morse et al., described how coxsacksackievirus B4 (CVB4) alters the microbiome and host-barrier function to accelerate TID in NOD mice. CVB4 infection induced rapid microbiome changes, erosion of the mucosal barrier, and increased gut bacterial translocation to the pancreatic lymph node. Transfer of fecal matter from CVB4-infected NOD mice increased the development of TID in female, but not male antibiotic-depleted NOD recipients. This increase was associated with reduced short chain fatty acid receptor expression and decreased IL-10-producing regulatory cells in the gut, suggesting the development of a less tolerogenic gut immune profile. These results provide insights into how enteroviruses may induce the development of TID and revealed a key sex difference in virus-mediated T1D development in mice.

## The immunoglobulin repertoire in endemic phemphigus foliaceus

Phemphigus foliaceus (PF) is an autoimmune disease that is characterized by development of autoantibodies against desmoglein-1 that disrupt adhesion between the upper layers of the skin and induce skin blistering. Although a very rare condition, in certain areas in Brazil it is endemic and can affect ~3% of the population (Calonga-Solis et al.). To address the molecular basis of this endemic autoimmunity, Calonga-Solis et al. sequenced the B cell receptor heavy chain and the variable regions of IgM and IgG of a limited number of PF patients and controls within the endemic region as well as controls from outside of the endemic region. Remarkably, both patients and controls living within the endemic region showed dramatically lower clonotype diversity suggesting that the immune system of these individuals is subject to intense environmental pressure. Compared to controls in the endemic region, BCRs of PF patients had increased IGHV3-30 usage and unique clusters of CDR3 sequences that may comprise the autoreactive B cell population. Future studies could use these cloned BCR sequences to identify the environmental trigger in endemic PF.

Overall, our Research Topic revealed new insights into the causal relationships and molecular mechanisms of how environmental factors can trigger autoimmunity.

## Author contributions

SD: Conceptualization, Writing – original draft, Writing – review & editing. JC: Writing – original draft, Writing – review & editing. JG: Writing – original draft, Writing – review & editing. MH: Writing – original draft, Writing – review & editing.
